# Supplementary material for: The interactome of intact mitochondria by cross-linking mass spectrometry provides evidence for coexisting respiratory supercomplexes
Source: Mol Cell Proteomics. 2017 Dec 8;17(2):216–32. doi: 10.1074/mcp.RA117.000470 (PMC5795388; doi:10.1074/mcp.RA117.000470)
Supplement: Supplemental Data [file supp_RA117.000470_133922_0_supp_23978_fzffwf.zip › spectra_annotation/mito_spectra_annotation/18-1-3-1-3-1.pdf]

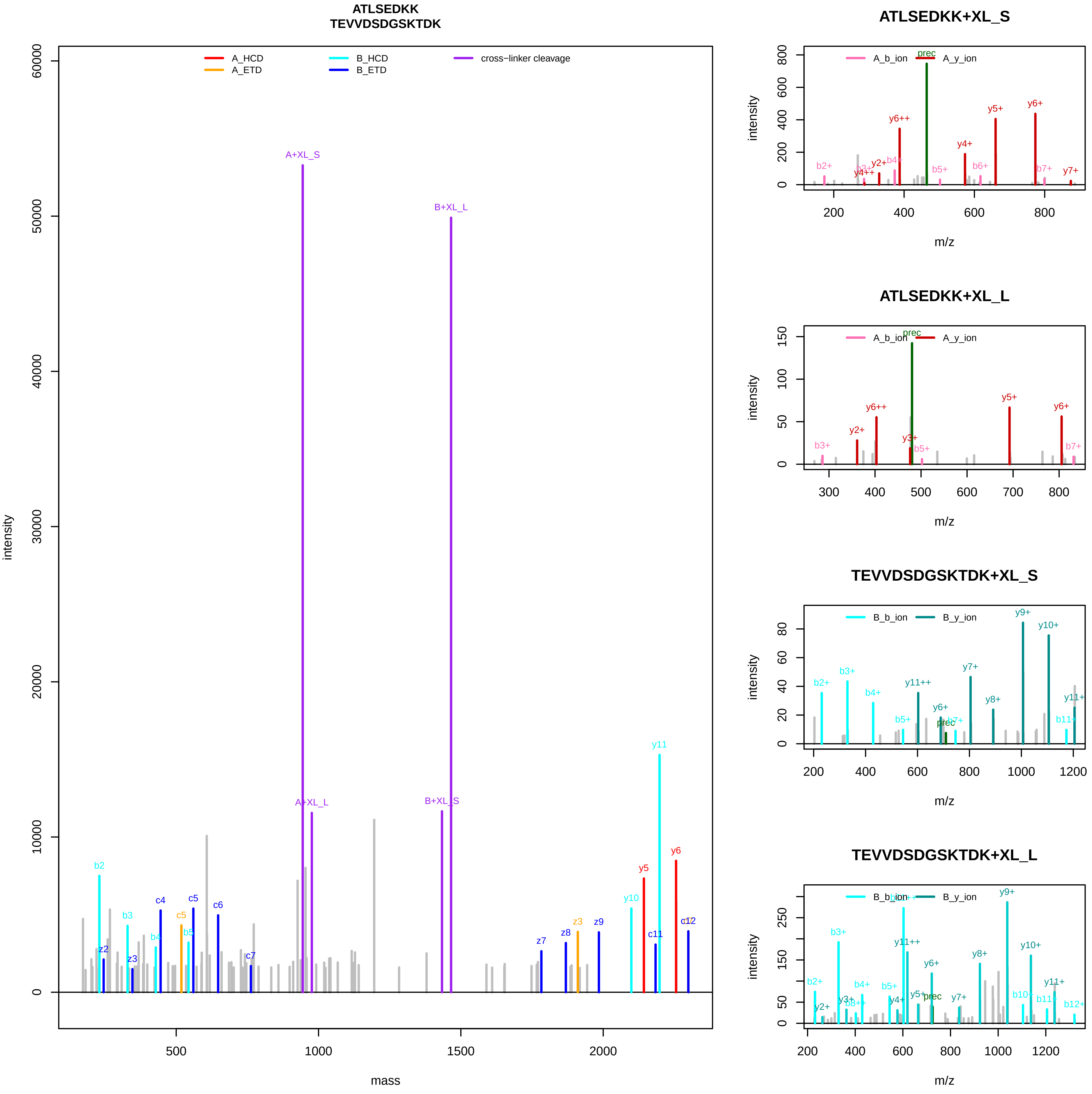

ATLSEDKK
TEVVDSDGSKTDK
A_HCD
A_ETD
B_HCD
B_ETD
cross−linker cleavage
intensity
mass
A+XL_S
B+XL_L
A+XL_L
B+XL_S
b2
b3
b4
b5
z2
z3
c4
c5
c6
c7
z7
z8
z3
z9
y10
y11
y5
y6
c11
c12
0
10000
20000
30000
40000
50000
60000
500
1000
1500
2000
ATLSEDKK+XL_S
A_b_ion
A_y_ion
prec
intensity
m/z
ATLSEDKK+XL_L
TEVVDSDGSKTDK+XL_S
B_b_ion
B_y_ion
TEVVDSDGSKTDK+XL_L
